# Knowledge, Attitude, and Practice of Pediatricians for Eye Care of Children: A Personal Survey in Saudi Arabia

**DOI:** 10.7759/cureus.69630

**Published:** 2024-09-18

**Authors:** Sultan Alzuhairy, Mashael Alsugair, Mayyaz Alqubays, Mohammed A Alzuhayri, Abdulaziz M Alsugair

**Affiliations:** 1 Ophthalmology, College of Medicine, Qassim University, Buraydah, SAU

**Keywords:** childhood blindness, children's, eyecare, kap, pediatricians

## Abstract

Purpose: To review the level, determinants, and sources of knowledge (K), attitude (A), and practice (P) of pediatricians regarding eye care of children in Saudi Arabia in 2024.

Methods: In this cross-sectional survey, 120 pediatricians from central Saudi Arabia were invited to participate in a one-on-one study. A questionnaire included demographic and work profiles, 20 questions on knowledge, five on attitude, and five on practices. Responses were graded using a Likert scale. Median scores were associated with independent variables.

Results: We surveyed 113 pediatricians. The excellent knowledge, attitude, and practice grades were 78.8%, 30.1%, and 41.6% of participants, respectively. The knowledge score was not associated with any demographic variable. The attitude score was associated with the younger age group (P = 0.03), seniority (P = 0.001), and higher knowledge score (P = 0.01). The practice score was associated with females (P = 0.02) and hospitals with eye departments (P = 0.001), who underwent eye care training (P = 0.004), knowledge score (P < 0.001), and A score (P = 0.001). Reading journals, attending conferences, and eye care workshops were current sources of knowledge. However, the desired sources were eye care, which should be included in the curriculum, Google and PubMed searches, and interactions with ophthalmologists.

Conclusions: Pediatricians' high level of knowledge of eye care is promising. However, based on the demography variations noted, their attitude and practices need strengthening. Policies for eye screening and referrals from pediatricians to ophthalmologists may be discussed in conferences, workshops, and policy documents. Knowledge should be disseminated using the modes preferred by pediatricians.

## Introduction

Children below 15 comprise 25% of the global and Saudi Arabian population in 2024 [1]. Visual impairment (VI) in children has a profound and lifelong negative impact on their overall development and quality of life. Therefore, it needs special attention compared to VI in adults. Global initiatives such as VISION 2020 and Universal Health within Millenium Development Goals have focussed and demonstrated great achievements in reducing eye ailments related to nutritional and infectious conditions, including children [2-4].

Pediatricians are the primary human resources and team leaders in child healthcare services. They provide primary eye care through preventive, promotional, and rehabilitative services. They diagnose red eye conditions and provide first-line treatment to children for common eye diseases. Eye screening at birth and red reflex testing in infants and children during immunization visits are standard protocols pediatricians follow for early detection of sight-threatening ailments. Child healthcare team members screen three- to five-year-old children either with the help of screening tools or by vision assessment that helps in early detection of strabismus, refractive error, amblyopia, and birth defects affecting vision. Pediatricians then refer the case to pediatric ophthalmologists or general ophthalmologists trained to treat eye problems in children [5-7]. The World Health Organization recommends member countries coordinate services within and across sectors for better eye care for children and better cooperation among pediatricians and ophthalmologists [8].

Exposure to eye care during undergraduate and postgraduate education in pediatrics is limited and not uniform. Integration of primary eye healthcare within primary health services is encouraged, but pediatricians are often distributed at primary health centers, general hospitals with pediatric departments, or pediatric hospitals. Therefore, their interaction varies, and updating their eye care skills is needed. Their knowledge about common and sight-threatening eye ailments, their attitude, and their practice in strengthening eye care of children are thus affected by several factors. They seek knowledge of current eye care in children using different available sources, such as attending eye workshops and conferences, and are often guided by pharmaceutical industries. Rapidly evolving Gulf countries have limited native child health professionals and have experts hired from different countries. They could follow different preferred patterns of practice (PPP) for eye care in children. Their practices, therefore, need to be standardized through protocols, and their activities must be monitored. The research on the knowledge (K), attitude (A), and practice (P) of pediatricians and their determinants helps strengthen children's eye care. Such surveys to determine KAP and its enablers are published in different countries [9-11]. There are also studies from researchers in Jordan and Saudi Arabia [9,12-14]. Hersi et al., in a survey of 51 Saudi pediatricians and 92 family physicians working with the National Guard hospitals of Saudi Arabia, 61.5% of them had a low level of knowledge about eye care, and 26.4% of pediatricians routinely did eye examinations [9]. A review of 29 published articles from 10 African countries noted that affordability, access, and availability of eye care to children are significant barriers for pediatricians [11]. A study of 105 pediatricians in Riyadh reported that 70% of pediatricians conduct eye assessments of children and refer 59% of premature children for retinopathy of prematurity screening [14]. Most of these studies were descriptive, and determinants of KAP and sources of knowledge were not studied. We present the level of KAP regarding eye care among Saudi Arabian pediatricians and their current and desired future models of seeking knowledge.

## Materials and methods

The research and ethical committee of Qaseem University approved this research (IRB#: 607/43/6833). We conducted this cross-sectional survey in June and July 2024. The study population included all pediatricians in the Qaseem region of Saudi Arabia. All 2,000 pediatricians working in the Qaseem region of Saudi Arabia, as reported by the Ministry of Health, were the study population for the study [15]. We included pediatricians registered with the Saudi Commission of Medical Specialities and practicing in the Qaseem region if they consented to participate in the survey. Those declining to participate, away from the Kingdom for further studies or on vacation during the survey, were excluded. For logistic reasons, we randomly selected six hospitals in the region and included all pediatricians in this hospital for the survey.

As a study in Kenya reported, we assumed that good eye assessment practices among pediatricians were in 70% of pediatricians [16]. To achieve a 95% confidence interval, an acceptable error margin of 10%, and a clustering effect of 1.5, we need to survey at least 120 pediatricians. We used the Stat calculator of OpenEpi software prepared by Emory University of the USA to estimate the sample size of a cross-sectional study [17]. The formula used for sample size calculation was n = [DEFF*Np(1-p)]/ [(d2/Z21-α/2*(N-1)+p*(1-p)].

The survey questionnaire was adopted based on other studies in Saudi Arabia and other countries [12,13,18]. The demographic information included age group, gender, type of workplace, category of pediatric practice, and years of pediatric experience. They also reported the kind of exposure to eye care training and the time of such training. The questionnaire for KAP on eye care contained 20 questions related to knowledge about eye ailments and their aetiologies, assessments, and treatments (Appendix 1, Table 4). Five questions assessed the attitude and practices of eye care among pediatricians. The responses were five grades on Likert scales ranging from strongly disagree, disagree, neutral, agree, and strongly agree. The survey tool underwent reverse translation as the participants used the Arabic questionnaire. A pilot was carried out on 10 health staff not included in the final data for analysis. The internal validity of responses to the knowledge, attitude, and practice questionnaire was high (Cronbach alpha = 0.843). The biostatistician performed a Likert scale analysis in line with the internationally recommended method for health studies [19]. The five grades of Likert scale responses were given a 1-5 score.

The data from Google Forms were transferred into the Statistical Product and Service Solutions (SPSS, version 25; IBM SPSS Statistics for Windows, Armonk, NY). Our study had a small subsample, and skew deviations were noted in outcomes in subgroups. Therefore, we used a nonparametric data presentation method and validation testing methods. The median response score of the knowledge group for the questions was calculated to note the overall knowledge score. A similar exercise was performed for attitude and practice groups of questions. We plotted graphs to visualize the five graded Likert scale responses on KAP. We used a parametric method to compare a subgroup's overall knowledge response score. For two sub-groups, we used the student's t-test. Two-sided P values were estimated. We used the chi-square test for more than two subgroups, and P values were noted. A p value of <0.05 was considered statistically significant.

## Results

We surveyed 113 pediatricians in this study. Table 1 describes the participants' demographics and work profiles. Three-fourths of the participants were residents, pediatricians under 30 years of age, and pediatricians working in a general hospital. The survey adequately represented participants of both genders. Only one in 10 participants had more than five years of childcare experience after qualification.

**Table 1:** Profile of pediatricians surveyed for knowledge, attitude, and practice for eye care in Saudi Arabia.

		Number	Percentage
Age-group	21 to 30	88	77.9
	31 to 40	17	15
	41 to 50	4	3.5
	> 50 years	4	3.5
Gender	Male	51	45.1
	Female	62	54.9
Seniority	Resident pediatrician	84	74.3
	Fellow	5	4.4
	fulltime staff	16	14.2
	Consultant	8	7.1
Place of work	General Hospital	86	76.1
	Hospital with eye department	17	15
	Private clinics	10	8.8
Years in child care	Less than two years	64	56.6
	2 to 5 years	35	31
	5 to 10 years	3	2.7
	More than ten years	11	9.7
Ophthalmic training	No	37	32.7
	During undergraduate studies	69	61.1
	After pediatric training	7	6.2

**Figure 1:**
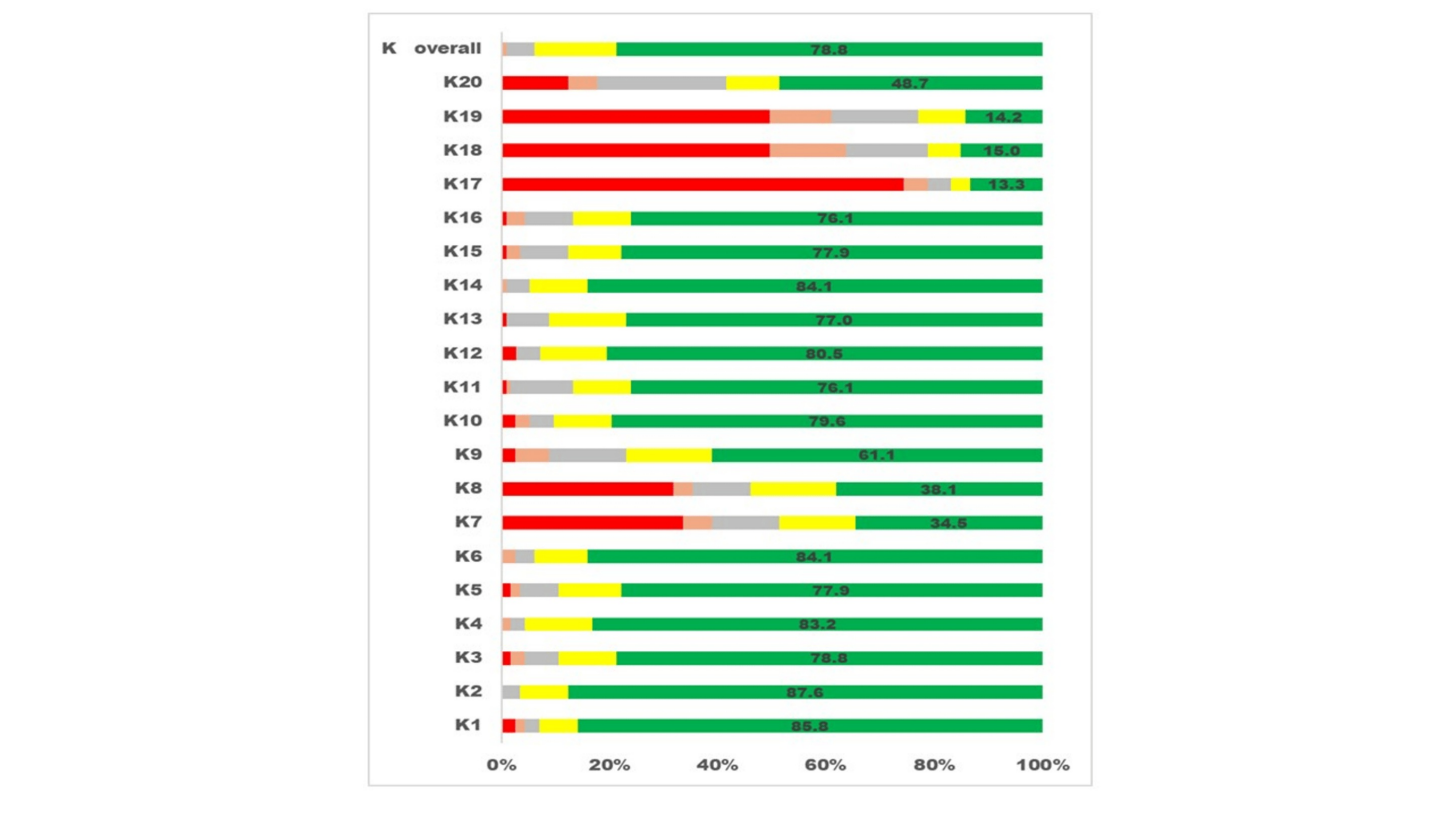
Visualising the five graded Likert scale responses to questions about vision and eye care of children related to "knowledge" by pediatricians. The X-axis displays the percentage proportion of participants giving a grade of response, and the Y-axis displays the overall knowledge.

Figure 1 presents the responses to the 20 knowledge questions and the participants' median scores for all knowledge-related questions.

It is worth noting that questions related to red eye and allergic conjunctivitis management received responses that reflected incorrect knowledge. Similarly, responses associated with the use of contact lenses and the role of opticians in the eye care of refractive error in children also showed less than desired knowledge.

**Figure 2:**
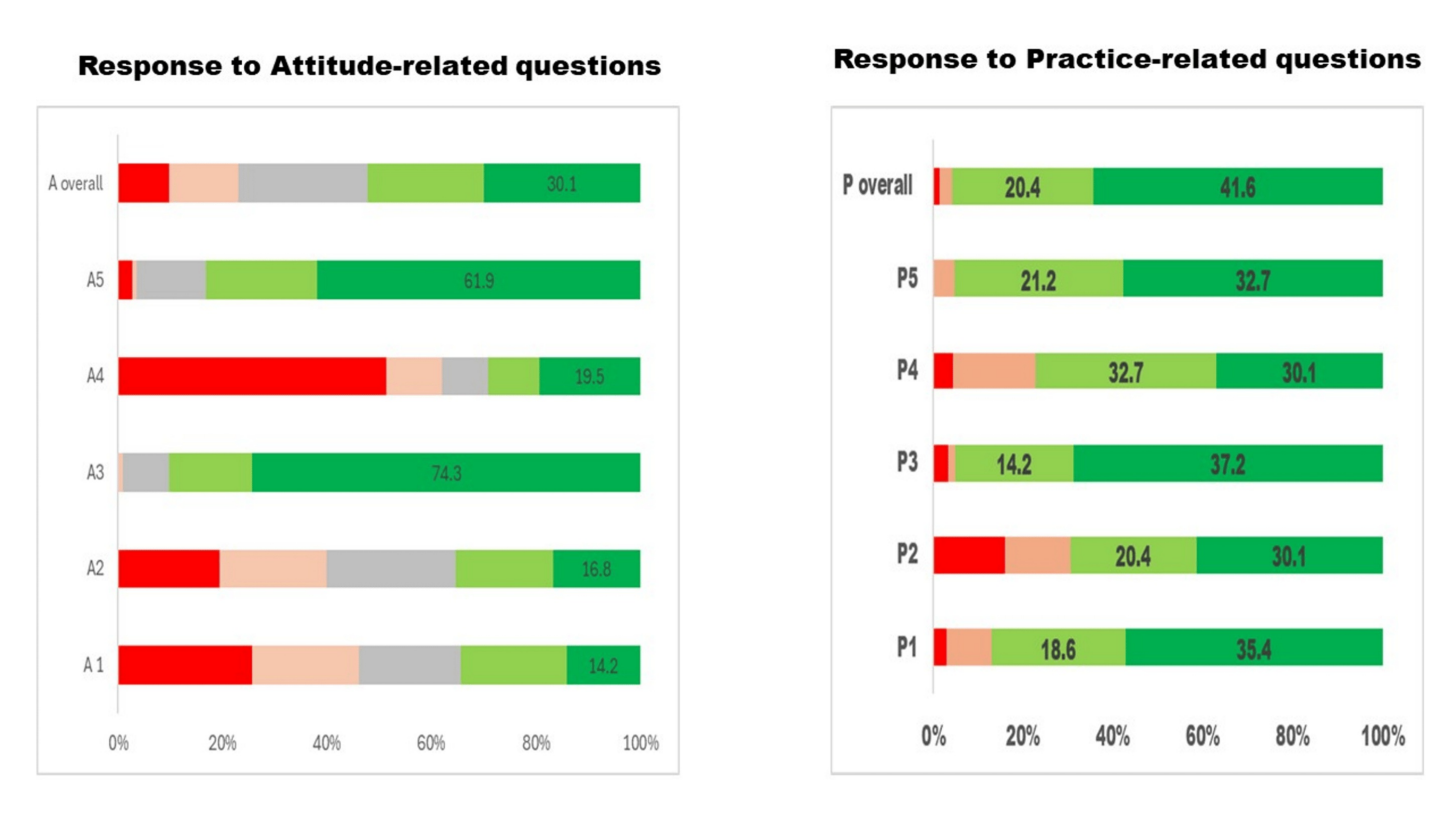
Visualising the five graded Likert scale responses to questions about vision and eye care of children related to "attitude" (A) and practice (B) by pediatricians. The X-axis displays the percentage proportions of participants with attitude and practice scores, and the Y-axis displays the questions related to attitude and practice for eye care and median scores of the total A & P scores.

Figure 2A illustrates the responses to the five attitude-related questions and the median score of participants for all attitude-related questions. It is encouraging to see a positive attitude towards teamwork and receiving feedback from ophthalmologists about the eye care of children under their care.

Figure 2B shows responses to the five practice-related questions and the participants' median scores for all practice-related questions. The attitude was positive for teamwork and receiving feedback from ophthalmologists about the eye care of children under their care. One-third of participants had excellent grade responses to the practice-related questions.

We associated demographic parameters with the median knowledge, attitude, and practice-related questions response score. Table 2 shows the chi-square and two-sided p values.

**Table 2:** Demographic and work-related determinants of the knowledge. Attitude and practice for eye care of children among pediatricians in Saudi Arabia.

	Knowledge		Attitude		Practice	
	Chi-square	P value	Chi-square	P value	Chi-square	P value
Age group	15.6	0.07	22.3	0.03	8.7	0.73
Gender	2.5	0.47	3.9	0.42	11.3	0.02
Workplace	6.8	0.34	9.4	0.31	26.6	0.001
Seniority	5.3	0.8	33.8	0.001	14.4	0.28
Exposure to eye care training	0.8	0.86	8.9	0.06	15.2	0.004
Knowledge score			23.4	0.02	46.5	<0.001
Attitude score					41.1	0.001

The K score was not associated with any demographic variable. The A score was associated with the younger age group (P = 0.03), seniority (P = 0.001), and higher knowledge score (P = 0.01). The P score was associated with females (P = 0.02) and hospitals with eye departments (P = 0.001) who underwent eye care training (P = 0.004), K score (P <0.001), and A score (P = 0.001).

The pediatricians replied about their current and desired sources in the future for seeking knowledge on eye care for children (Table 3).

**Table 3:** Current and desired future sources of eye care knowledge among pediatricians.

	Current sources of knowledge		Desired sources of knowledge	
	Number	Percentage	Number	Percentage
During undergraduate studies	0	0.0	74	65.5
During post-graduation studies	3	2.7	53	46.9
Attending training workshop on eye care	24	21.2	16	14.2
Attending conference	37	32.7	19	16.8
Reading journal articles	49	43.4	36	31.9
Textbooks	0	0.0	33	29.2
Social media	0	0.0	19	16.8
Google and PubMed	0	0.0	33	29.2
Interaction with ophthalmologists	0	0.0	14	12.4

There is a significant difference between the current and preferred sources of eye care knowledge. Reading journals, attending conferences, and eye care workshops were current sources of knowledge. However, the desired sources were "inclusion of eye care in the curriculum," Google and PubMed searches, and interactions with ophthalmologists.

## Discussion

Three-fourths of pediatricians had excellent knowledge about issues related to eye care for children. Only one-third had a positive attitude toward eye care, and less than half had excellent practices for eye care in children. The pediatrician's age and seniority influenced the positive attitude toward eye care of children. The eye care practices of pediatricians were better in females, those working in hospitals with eye departments, those with eye care training, and those with better knowledge and a positive attitude. One in 15 pediatricians had eye care training after pediatric education. Survey participants desired eye care inclusion in the curriculum and access to knowledge through journals on the Internet.

In this study, pediatricians' perceived knowledge, attitudes, and practices regarding eye care in Saudi Arabia are vital for reviewing children's eye care and ways to strengthen eye care and pediatricians' contribution to attaining the Global and Saudi Arabia VISION 2030 goals of Universal Health. An objective assessment of eye services to children at primary healthcare and institutions of pediatric care may complement present study outcomes to act upon. The factors affecting attitude and practices could be used while strategizing the training, disseminating knowledge, and generating indicators to monitor children's eye care. Face-to-face surveys and using Google Forms to note responses ensured quick and reliable responses with minimum bias.

The knowledge about eye care was of excellent grade in 78.8% of pediatricians. In a study in the neighboring country, Jordan, Ababneh et al. studied 48 participants and reported satisfactory levels of knowledge [12]. Regassa et al. surveyed 79 pediatricians in Ethiopia and found that eye care knowledge needed better quality [19]. In another African country, Kenya, 70% of surveyed pediatricians had good eye care knowledge for children [16]. Our study findings matched Almazrou et al.'s findings, who reported an excellent understanding of most eye ailments except retinopathy of prematurity among pediatricians of Saudi Arabia [13]. In our study, knowledge about detecting refractive error, optometrists' role, and managing red eye and allergic conjunctivitis was less than desired. In eye care training workshops for staff related to childcare, the facilitators should include these components to impart correct information.

In the present study, 30% of pediatricians surveyed had an excellent attitude toward child eye care. Areas with promising attitude outcomes were teamwork among ophthalmologists and childcare for newborns and infants. The feedback from ophthalmologists regarding further follow-up and care of children with eye problems was less than desired. Changing attitudes takes longer but can be achieved by imparting correct information through reliable and desired sources. The display of success stories changes attitudes [20].

Pediatricians perceived eye care practices for children mainly as focussed on their actions in liaison with ophthalmologists, and the study suggested that four in 10 participants had excellent grades for eye care practices. Most previous studies also noted eye care practices less than the desired level among pediatricians [12,13,16,19]. The factors influencing good practices must be identified and used while revising the strategies to impart knowledge, change attitudes, and improve eye care practices. Taking the help of ophthalmologists to strengthen the management of sight-threatening conditions is welcome, but always seeking eye care professionals' help shows a lack of training and protocols for primary eye care for children. These frequent references may encourage parents to approach care professionals directly and increase the burden of secondary and tertiary eye care services with trivial cases. Receiving ophthalmologist feedback on each case referred is preferred but may only sometimes be feasible. For a child that needs joint management of eye and systemic ailment, it might be ideal to have a report to childcare professionals to take further action and follow up on the schedule. The focus of pediatricians on liaising with parents to implement preventive eye care, as noted in 41% of pediatricians, is promising but needs further improvement [21].

In this study, we noted that pediatricians' knowledge and attitudes toward child eye care influence their eye care practices. By improving their knowledge, we expect to strengthen children's eye care and encourage more active participation of pediatricians in Saudi Arabia. The pediatricians recommended workshops and educational programs to disseminate knowledge on eye diseases and current practice patterns [13].

Most participants stated that, during their undergraduate and postgraduate studies, they learned about eye diseases in children and their management. However, only some had attended eye care training workshops and conferences after qualifying in pediatrics. In hospitals with the ophthalmic department, joint case discussion meetings for child health and eye health professionals could result in better interaction and dissemination of current trends in eye care to pediatricians. A session on eye care of children in pediatric conferences may be encouraged to present their experiences and teamwork with ophthalmologists. A wide gap between current and desired sources of knowledge about eye care among pediatricians is noted in the present study. This prompts the provision of modern tools for imparting eye care-related information, such as free access to medical journals, standard operating procedures, and frequent interaction between pediatric ophthalmologists and child healthcare providers. Periodic refreshing courses in eye care for pediatricians as part of capacity building the existing health staff could also be organized.

There were a few limitations in the present study. The recruitment marginally fell short of the desired sample size. Their recruitment was also not random and stratified. Seven pediatricians declined to complete the survey because they were leaving the country. Therefore, we could analyze only 113 participants' responses, which could marginally affect the survey outcomes. If all seven declining were considered poor or excellent knowledge, the awareness level would range between 65% and 76%. Therefore, one should do extrapolation of study outcomes with caution. The interaction with eye care professionals and practice patterns of pediatricians working in general hospitals with and without an ophthalmology department would differ in Saudi Arabia. Therefore, child healthcare provided in primary health centers and private clinics may be different and needs adequate representation of pediatricians of these subgroups.

In 2020, the Ministry of Health reactivated vision and eye screening for first- and fourth-grade children at primary healthcare and urged parents to seek these services for their wards [22]. Such screening was recommended by the expert committee members of the World Health Organization - Eastern Mediterranean region, with an example of Oman school vision and eye screening program operational annually since 1993 [23,24]. Understanding the need for early detection and standard management of retinopathy of prematurity, the guidelines for all related to preterm babies' health, including neonatologists and pediatricians, were published in Saudi Arabia in 2018 [25]. Although Saudi Arabia included hearing screening of newborns in their national newborn screening initiative in 2016, the program does not introduce a red reflex test recommended for eye screening of newborns [26,27].

The American Academy of Pediatrics recommends visual system assessment. However, they are not applied to infants and preschoolers in Saudi Arabia as a universal screening program [7]. Given the above challenges of early detection of eye and vision-related issues in children, pediatricians in Saudi Arabia have a significant and challenging role in early detection and reference to ophthalmologists. They should be involved in decision-making to introduce universal eye and vision screening guidelines to strengthen child healthcare. Their inclusion will be more effective if their knowledge and involvement are surveyed, and they are part of committees to revise screening programs to include eye ailments at different stages of children's growth. To achieve the 2030 Universal Eye Health Goals in Saudi Arabia, focusing on eye care for the pediatric age group, which comprises nearly 50% of the Saudi population, is urgently needed [28,29]. With the expansion of pediatric eye care in all regions of the Kingdom, better linkage of child healthcare providers and specialist ophthalmologists and eye screening and referral system from pediatricians to subspecialist ophthalmologists through policymaking and increasing awareness of care providers and community is needed.

## Conclusions

The high level of knowledge about childhood eye ailments among pediatricians noted in the present study suggests that knowledge alone does not translate into a positive attitude and practice of child healthcare in Saudi Arabia. Policies for eye screening and referrals from pediatricians to ophthalmologists may be discussed in conferences, workshops, and policy documents. The curriculum revision of pediatric and undergraduate medical studies could include child eye healthcare issues and standard operating procedures for referring children with eye problems to pediatric ophthalmologists. We also recommend similar studies to healthcare providers related to child health in other regions, nurses, and family physicians and have better interaction with pediatric ophthalmology units in the catchment areas. In addition to providing sources of knowledge per their preferences, introducing internationally approved protocols for early detection of eye and vision screening of children at different ages, strict monitoring of their implementation, and sharing the outcomes with pediatricians could motivate them to actively contribute to changing attitudes and practices.

## Appendices

**Table 4:** Survey questions related to the knowledge, attitude, and practice of pediatricians for eye care of children in Saudi Arabia.

K	Knowledge related questions	I strongly agree	I agree	Neutral	I disagree	I strongly disagree
1	All preterm babies must be screened by a pediatric ophthalmologist to rule out retinopathy of prematurity.					
2	At birth, the attending nurse and neonatologist must examine eyes for birth defects, white reflex, and nystagmus					
3	A 3—to 5-year-old child should be assessed for vision and eyes even if there are no symptoms or signs.					
4	All children must be periodically examined for refractive error in schools					
5	Children who are not improving their vision with corrections should be offered low-vision rehabilitation services in the school.					
6	A child with crossed eyes must be treated by an eye professional as soon as possible.					
7	Red-eye with discharge should be treated by a pediatrician.					
8	Red eye with itching should be treated by an ophthalmologist.					
9	Retinoblastoma: a childhood ocular malignancy should be suspected if another family member has a similar history.					
10	A child with ocular trauma should be referred immediately to an ophthalmologist and no ointment should be applied					
11	Indiscriminate use of steroid eye medication in children can cause blindness.					
12	A child should have restricted ‘screen time’ as it causes eye problems like myopia, dry eyes, etc					
13	A child should be encouraged to do more outdoor activities to prevent myopia.					
14	Vitamin A deficiency causes eye signs and night blindness.					
15	Vaccination against Measles reduces the risk of corneal blindness.					
16	The child advised to use spectacles must always comply.					
17	Contact lenses used by adolescent children can be shared.					
18	A qualified optometrist can treat eye diseases in Saudi Arabia.					
19	Getting spectacles for children prescribed by an optical shop is acceptable.					
20	Too much smartphone and computer use spoil the vision of children.					
A	Attitude related questions					
1	Eye screening and care should not be the responsibility of pediatricians.					
2	Pediatricians are adequate in treating children's eye problems.					
3	Eye care for preterm and newborn babies requires multispecialty teamwork.					
4	The crossed eye and refractive errors in young children need not be treated till they become older.					
5	Ophthalmologists should liaise with the pediatrician after assessing pediatric eye problems.					
P	Practice related questions					
1	I regularly refer my patients to ophthalmologists.					
2	I regularly receive feedback from ophthalmologists about children referred.					
3	I refer children with systemic problems to rule out ophthalmic manifestations.					
4	I regularly read new updates on eye care for children.					
5	I encourage parents to ensure preventive measures to protect the eyesight of children.					

## References

[1] (2024). Global and Saudi Arabia population 2024. Saudi Arabia population.

[2] Khandekar R (2017). Sustainable Developmental Goals (SDGs) and eye health care in Nepal. Nepal J Ophthalmol.

[3] Tan RJ, Sharma IP, Lucero-Prisno DE 3rd (2021). Childhood blindness: beyond VISION 2020 and the COVID-19 pandemic. Glob Pediatr Health.

[4] Philippin H, Morny EK, Heinrich SP (2024). [Global ophthalmology: update]. Ophthalmologie.

[5] Bonini S (2021). The red eye. Eur J Ophthalmol.

[6] Toli A, Perente A, Labiris G (2021). Evaluation of the red reflex: an overview for the pediatrician. World J Methodol.

[7] Donahue SP, Nixon CN (2016). Visual system assessment in infants, children, and young adults by pediatricians. Pediatrics.

[8] (2024). World report on vision. https://www.who.int/publications/i/item/9789241516570.

[9] Hersi RM, Naaman NK, Alghamdi AM, Alnahdi WA, Bukhari ZM, Almarzouki HS (2023). Knowledge and attitude toward eye disorders in children among pediatricians and family physicians: a survey study. BMC Ophthalmol.

[10] Tuğan BY, Sönmez HE, Yüksel N (2023). Knowledge, attitude, and practice toward eye disorders among pediatricians in Turkey. Pediatrician.

[11] Alrasheed SH (2021). A systemic review of barriers to accessing paediatric eye care services in African countries. Afr Health Sci.

[12] Ababneh LT, Khriesat W, Dalu SA, Hanania RJ, Ababneh BF, Nama’A BA, Jahmani T (2021). Knowledge of and attitude to eye disorders among pediatricians in North Jordan. Ann Med Surg (Lond).

[13] Almazrou AA, Aladawi AM, Alswayed S, AlSuhaibani R, Abukhaled J, Alqahtani N, Alz N (2024). Knowledge and attitude toward eye disorders among pediatricians in Saudi Arabia. IJMDC.

[14] Alshehri A, Alarfaj G, Kofi M (2020). Knowledge of primary care physicians regarding eye trauma among children under age 14 attending PHC, Riyadh, Saudi Arabia. Int J Adv Community Med.

[15] (2024). Part 2: health resources 2023. Book.

[16] Wanyama SP (2013). Knowledge, Attitude and Practice of Eye Diseases in Children Among Pediatricians in Kenya. UoN Digital Repository.

[17] Dean AG, Sullivan KM, Soe MM (2024). Open source epidemiologic statistics for public health. https://www.openepi.com/Menu/OE_Menu.htm.

[18] Regassa TT, Daba KT, Fabian ID, Mengasha AA (2021). Knowledge, attitude and practice of Ethiopian pediatricians concerning childhood eye diseases. BMC Ophthalmol.

[19] Harpe SE (2015). How to analyze Likert and other rating scale data. Curr Pharm Teach Learn.

[20] Grol R, Wensing M (2020). Effective implementation of change in healthcare: a systematic approach. Improving Patient Care: The Implementation of Change in Health Care, Third Edition.

[21] Keel S, Evans JR, Block S (2020). Strengthening the integration of eye care into the health system: methodology for the development of the WHO package of eye care interventions. BMJ Open Ophthalmol.

[22] (2024). MOH activates school-based screening program through healthcare centers. https://www.moh.gov.sa/en/Ministry/MediaCenter/News/Pages/News-2021-03-14-008.aspx.

[23] (2024). Guidelines for school eye health for the Eastern Mediterranean region (EMR). https://www.pbunion.org/IMPACT-EMR-Guidelines-1.pdf.

[24] Khandekar R, Gogri U, Al-Harby S (2018). Changing trends in myopia among schoolchildren in Oman: screening information over 11 years. Oman J Ophthalmol.

[25] Al Amro SA, Al Aql F, Al Hajar S, Al Dhibi H, Al Nemri A, Mousa A, Ahmad J (2018). Practical guidelines for screening and treatment of retinopathy of prematurity in Saudi Arabia. Saudi J Ophthalmol.

[26] Gosadi IM (2019). National screening programs in Saudi Arabia: overview, outcomes, and effectiveness. J Infect Public Health.

[27] Malik AN, Evans JR, Gupta S, Mariotti S, Gordon I, Bowman R, Gilbert C (2022). Universal newborn eye screening: a systematic review of the literature and review of international guidelines. J Glob Health.

[28] (2024). Health and well-being profile of the Eastern Mediterranean region. https://iris.who.int/handle/10665/348133.

[29] Rahman R, Qattan A (2021). Vision 2030 and sustainable development: state capacity to revitalize the healthcare system in Saudi Arabia. Inquiry.

